# H^+^-Independent Glutamine Transport in Plant Root Tips

**DOI:** 10.1371/journal.pone.0008917

**Published:** 2010-01-27

**Authors:** Huaiyu Yang, Martin Bogner, York-Dieter Stierhof, Uwe Ludewig

**Affiliations:** 1 Plant Biomass and Nutrition, Institute of Botany, Darmstadt University of Technology, Darmstadt, Germany; 2 Plant Physiology, Center for Molecular Biology of Plants (ZMBP), University of Tübingen, Tübingen, Germany; 3 Microscopy Unit, Center for Molecular Biology of Plants (ZMBP), University of Tübingen, Tübingen, Germany; University of Heidelberg, Germany

## Abstract

**Background:**

Glutamine is one of the primary amino acids in nitrogen assimilation and often the most abundant amino acid in plant roots. To monitor this important metabolite, a novel genetically encoded fluorescent FRET-reporter was constructed and expressed in *Arabidopsis thaliana*. As a candidate for the glutamine fluxes, the root tip localized, putative amino acid transporter CAT8 was analyzed and heterologously expressed in yeast and oocytes.

**Principal Findings:**

Rapid and reversible *in vivo* fluorescence changes were observed in reporter-expressing root tips upon exposure and removal of glutamine. FRET changes were detected at acid and neutral pH and in the presence of a protonophore, suggesting that part of the glutamine fluxes were independent of the pH. The putative amino acid transporter CAT8 transported glutamine, had a half maximal activity at ∼100 µM and the transport was independent of external pH. CAT8 localized not only to the plasma membrane, but additionally to the tonoplast, when tagged with GFP. Ultrastructural analysis confirmed this dual localization and additionally identified CAT8 in membranes of autophagosomes. Loss-of function of CAT8 did not affect growth in various conditions, but over-expressor plants had increased sensitivity to a structural substrate analog, the glutamine synthetase inhibitor L-methionine sulfoximine.

**Conclusions:**

The combined data suggest that proton-independent glutamine facilitators exist in root tips.

## Introduction

Amino acid uptake from the soil by plant roots can play a significant role in ecosystems with low mineralization rates [1]. While plants generally have the capacity to take up many amino acids, these often negatively interfere with biomass production and even inhibit growth [2]. The model plant *Arabidopsis* has the capacity to acquire and utilize a number of amino acids for growth, including the amide glutamine (Gln) [3].

Transporters for the uptake of amino acids from the soil have been molecularly identified. The lysine-histidine transporter 1 (LHT1) is the major high affinity transporter in *Arabidopsis* and imports a broad spectrum of uncharged and acidic amino acids [4], [5]. Cationic amino acids, such as lysine, are poor substrates of LHT1, but are imported by amino acid permease 5 (AAP5) [6], a transporter that efficiently transports cationic amino acids when expressed in oocytes and yeast [7]. Furthermore, the general amino acid permease 1 (AAP1) was identified to participate in amino acid uptake in the low affinity range [8] and CAT6 also appears to be involved in amino acid uptake [9]. All these transporters are proton coupled transport systems that accumulate amino acids from the acidic apoplast into the cytosol [4], [7]. Classical analyses on plant plasma membrane vesicles provided strong evidence that the transport of amino acids is driven by the pH-gradient [10].

In addition to the uptake, significant efflux of selected amino acids occurred from roots to supply microorganisms in the soil [11]. In *Arabidopsis*, efflux of Gln from roots may occur [6]. Metabolite and nutrient efflux is prominent from transfer cells, and is likely mediated by molecularly distinct transport systems, such as H^+^-independent facilitators [12]. A bi-directional amino acid transporter has been recently identified in *Arabidopsis* [13]. Efflux systems have considerable roles in vascular unloading and metabolite/nutrient unloading at the maternal/filial interface in seeds [14]. Proton-independent sugar facilitators have recently been molecularly identified that facilitate sugar efflux in legume seed coats [15]. Such pH-independent transporters/channels are likely to exist also for amino acids, but have not yet been molecularly identified.

In contrast to the situation at the plasma membrane, little is molecularly known about amino acid transporters at the tonoplast. The putative amino acid transporter CAT2 has been localized to the tonoplast, when tagged with GFP and transiently expressed in protoplasts [16]. This localization of CAT2 was confirmed in a proteomic study [17], where also CAT4 was identified in the vacuolar proteome. In another study, CAT2, CAT4, CAT8, and CAT9 were identified in the vacuolar proteome [18]. However, CAT8 localized mainly to the plasma membrane when expressed in protoplasts, although it occasionally occurred additionally in the tonoplast [16]. Vacuolar concentrations of most amino acids are similar to or lower than the cytosolic concentrations in many plants [19]. Due to the large vacuolar lumen, the total amino acid content in the vacuole is often higher than that in the cytosol, although the concentration is equal or lower [20].

Fluorescent reporters of several metabolites have been recently developed and were used to monitor fluxes within intact mammalian or plant cells [21], [22]. Similar reporters for arginine were expressed and used in plants to detect amino acid influx [23].

In this study, further fluorescent FRET-reporters were developed, which were most sensitive to glutamine *in vitro*. The fluorescence of plants expressing a reporter in root tips changed upon the external application of glutamine. The fluorescent signals indicated that part of the Gln fluxes in root tips were proton independent. Previous functional analysis in yeast and the localization in the root tip suggested that the putative amino acid transporter CAT8 might be involved in the glutamine fluxes. CAT8 was identified as a H^+^-independent Gln transporter using yeast and oocytes as heterologous hosts. GFP-tagged CAT8 labeled the plasma membrane, as well as the tonoplast. CAT8 over-expression resulted in higher sensitivity to the toxic structural Gln/Glu analog L-methionine sulfoximine (MSX).

## Results

### Development of Fluorescent Glutamine Reporters

**Figure 1:**
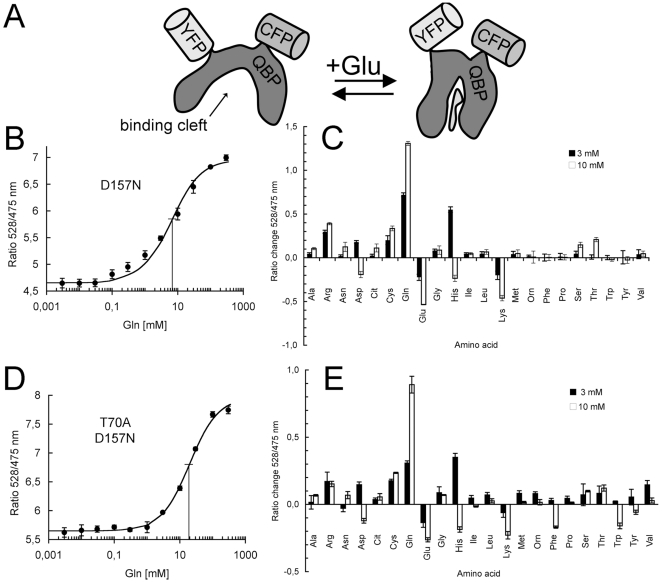
Construction of a FRET reporter for glutamine and *in vitro* properties of the purified protein. (A) Schematic design of the reporter. (B,C) FRET reporter with mutation D157N. (B) K_d_^app^ = 6.9 mM Gln, dynamic range *r*_max_−*r*_min_ = 2.21 (n = 3). (D) Specificity to all proteinogenic amino acids. Ratio change upon addition of 3 mM (black bars) or 10 mM (white bars) of the amino acids given in tree letter code (n = 3). (D,E) FRET reporter with mutations D157N and T70A. (D) K_d_^app^ = 18.8 mM Gln, dynamic range *r*_max_−*r*_min_ = 2.31 (n = 3). (E) Specificity and ratio change upon addition of 3 mM (black bar) or 10 mM (white bar) of the amino acids given in tree letter code (n = 3).

Several fluorescent reporters for metabolites have recently been developed, which transfer substrate-specific conformational changes of a ligand-binding domain into fluorescence ratio changes of attached chromophores [21] (Figure 1A). In this study, the bacterial glutamine binding protein (QBP) from *E.coli* was chosen as the specific ligand-binding domain [24]. In a previous study, the attachment of chromophores to this QBP binding domain rendered the resulting chimeric protein sensitive to arginine, but not to glutamine [23]. That arginine reporter construct was now modified by mutations in the ligand-binding pocket and shortened in the linker between the chromophores and QBP. The mutations were chosen according to mutational work on mammalian ionotrophic glutamate receptors, which resemble the binding domain of QBP in structure and sequence [25]. An aspartate (to asparagine) and/or a threonine (to alanine) in the ligand-binding pocket were exchanged. These selected residues are in close contact with the substrate [23], [24].

By these modifications, chimeric reporters were obtained that responded to millimolar glutamine (Figure 1). For the construct containing the single exchange of aspartate 157 to asparagine (D157N) in QBP, an apparent K_d_ = ∼6 mM was determined. The FRET ratio changes in the construct containing the additional mutation T70A had an about 3-fold lower affinity and were saturated at an apparent K_d_ = 18.8 mM for Gln (Figure 1B,D). The specificity of the fluorescence changes was then determined for all proteinogenic amino acids at concentrations of 3 mM and 10 mM (Figure 1C,E). The fluorescence of both constructs was reversibly affected by a number of amino acids, but Gln had the largest effect on the total FRET ratio.

### In Vivo Imaging of Cytosolic Gln in Plant Root Tips

**Figure 2:**
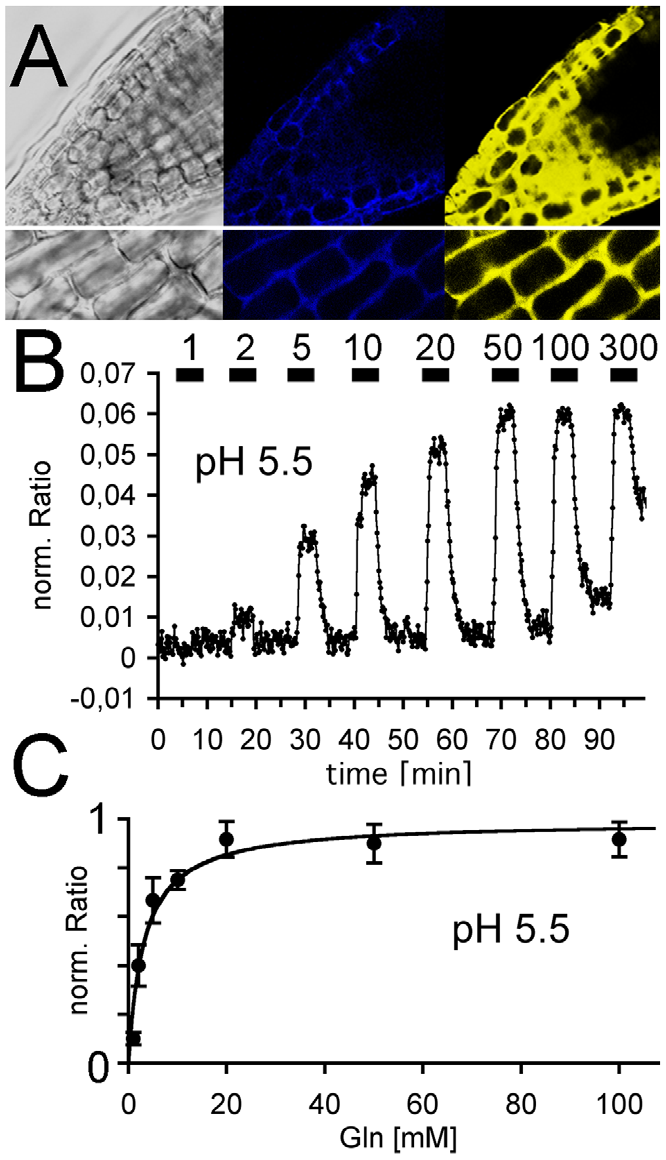
*In vivo* function of the Gln (D157N) reporter in *rdr6* plants. (A) Confocal snapshots of a responding root tip (gray) with blue (CFP) and yellow (YFP) fluorescence. Higher magnification pictures are shown in the lower panels. (B) Normalized fluorescence ratio changes upon superfusion with Gln-containing nutrient solution of *Arabidopsis rdr6* root tips. Numbers indicate Glutamine concentrations in mM. (C) Apparent saturation kinetics of the Ratio changes revealed a K_d_ of 8.5 mM.

The reporters were transferred using *Agrobacterium*-mediated transformation to *Arabidopsis* and the expression was driven by the 35S promoter from cauliflower mosaic virus. The fluorescent signal from transformed plants was, however, too low for reliable imaging and decreased in subsequent generations. Therefore, the reporters were stably expressed in the *rdr6* background that lacks part of the RNA silencing machinery. *rdr6* plants have been successfully used to monitor sugar metabolites with similar reporters [22], [26]. Using an inverted microscope with optimized shutter and filter system for the used FRET pair, fluorescence changes upon glutamine (Gln) superfusion were visible in reporter-expressing plants. The signals were strongest in root tips, which were selected for further metabolite imaging (Figure 2). After initial screening for plants with strong fluorescence, the concentration-dependent FRET changes upon Gln were continuously monitored (Figure 2). The largest and most reliable FRET ratio changes were observed with the D157N-based reporter, which was therefore used for all subsequent experiments. The signals from the two chromophores in a Gln-responding root tip are shown as confocal images in Figure 2A. The fluorescence was excluded from the lumen of the vacuoles and was mainly cytoplasmic. In subsequent recordings, a slow total fluorescence decrease was observed that resulted from chromophore bleaching, even without any external application of amides. All traces shown were therefore corrected for this continuous bleaching effect.

The fluorescent ratio was recorded every ten seconds and rapidly increased after application of Gln (Figure 2B). After the signal had reached a constant steady-state value, the Gln was rapidly removed by continuous superfusion. This was accompanied by a rapid decrease to the initial FRET levels before Gln application. This decrease was only 3–10-fold slower than the time to steady state after addition of Gln. This rapid washout was not limited by the solution flow within the recording chamber, because only tens of seconds were required for complete exchange of the solutions. The FRET decrease must therefore reflect the decreased cytosolic steady-state concentration, which results from the combined effects of rapid metabolism, efflux and/or transient storage. The FRET response saturated with repeated superfusion of high levels of Gln (Figure 2B) and was slightly reduced at very high Gln, suggesting that saturable cellular Gln pools, such as the vacuoles, may buffer the cytosolic steady state to some extent. A few other amino acids than Gln were also tested, but did not elicit significant fluorescent responses, in accordance with the *in vitro* specificity of the reporter.

The maximal ratio change was plotted against the external concentration and could be fitted to a single-site binding isotherm with an apparent half maximal concentration (K_d_) of 8.5 mM Gln (Figure 2C). This value represented the external concentration at which the cytosolic Gln corresponded to the K_d_ of the purified reporter. Thus, after reaching a steady state with superfusion of 8.5 mM external Gln, the fluorescence of the cytosolic reporter was half maximally saturated (at its *in vitro* K_d_ of 6 mM). In principle, this value may reflect the saturation of the uptake systems for Gln and/or the saturation of the cytoplasmic Gln levels. The cytosolic Gln concentration had been determined in earlier studies by other methods and was typically in the low mM range, but depended on the nitrogen supply [19], [27].

**Figure 3:**
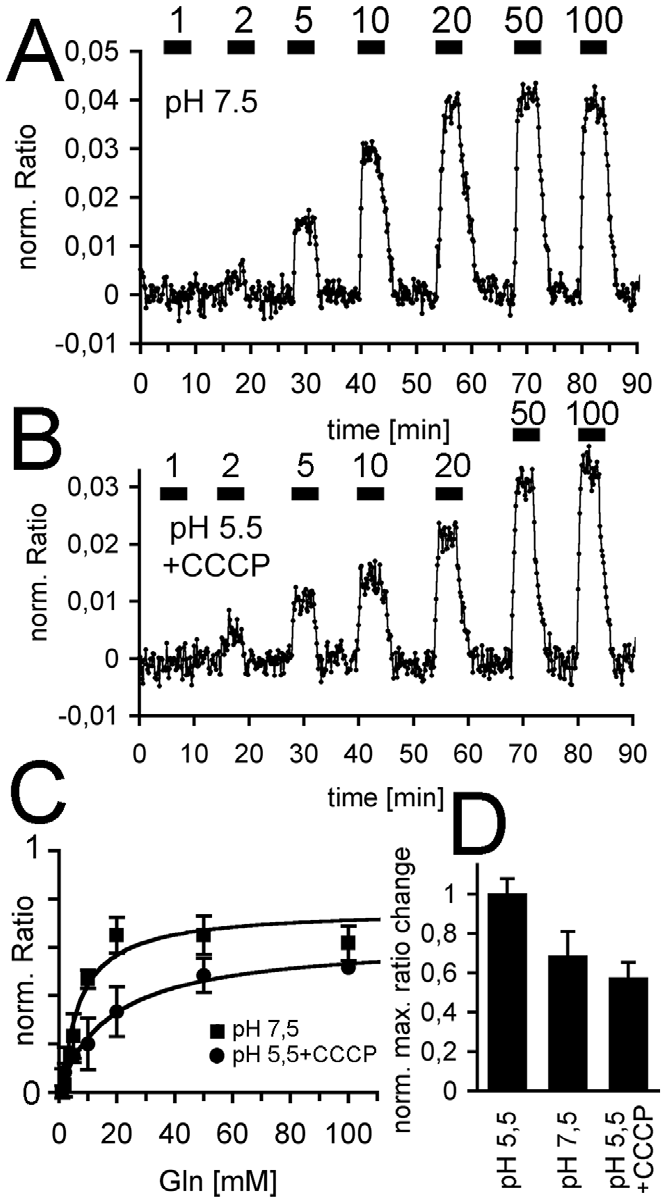
Similar Gln transport at pH 7.5 and partial reduction by a protonophore. (A) Fluorescence changes that correspond to an influx of Gln at pH 7.5. Superfusion by Gln (in mM) is indicated by the black bars. (B) Fluorescence changes in the presence of the protonophore CCCP (100 µM). Gln is given at times marked with black bars. Numbers indicate Glutamine concentrations in mM. (C) Concentration dependence of the fluorescence. Half maximal ratio change was K_d_^app^ = 8.2 mM (n = 2) at pH 7.5 (closed squares) and K_d_^app^ = 17.9 at pH 5.5 in the presence of CCCP (100 µM, n = 3, closed circles). (D) Maximal ratio changes at pH 7.5 and pH 5.5 with CCCP relative to those at pH 5.5.

The fluorescence ratio changes with Gln were then recorded with a solution that was buffered to pH 7.5 (Figure 3A). Again, FRET changes were recorded. These were of smaller amplitude then those at pH 5.5 (Figure 2), but were similarly reversible after withdrawal of the substrate. The relatively strong signal at pH 7.5 (Figure 3) was surprising, as all amino acid transporters characterized from plants so far were H^+^-coupled systems and only a small, residual transport activity was expected at pH 7.5. A fraction of the Gln influx that was detected by the fluorescent reporters may therefore have been carried by novel, pH-independent transporters or channels. This possibility was further supported by the observation that the Gln-dependent fluorescence was even observed in the presence of the protonophore CCCP (Figure 3B). The effectiveness of the uncoupler on membrane potential-dependent transport was tested in independent control experiments, but when this drug was applied at pH 5.5, the magnitude of the *in vivo* response was still large enough for reliable recording (Figure 3B,D). CCCP itself slightly affected the FRET ratios, consistent with a small change in the cytoplasmic pH or the ion concentrations [22]. The responses at pH 7.5 and with CCCP (pH 5.5) saturated with a half maximal external Gln concentration of ∼8 and 18 mM, respectively (Figure 3C). The presence of significant Gln fluxes at neutral pH and in the presence of a proton uncoupler may suggest the existence of a proton–independent Gln transporter.

### The Root Tip-Localized CAT8 Transported Gln in Heterologous Systems

**Figure 4:**
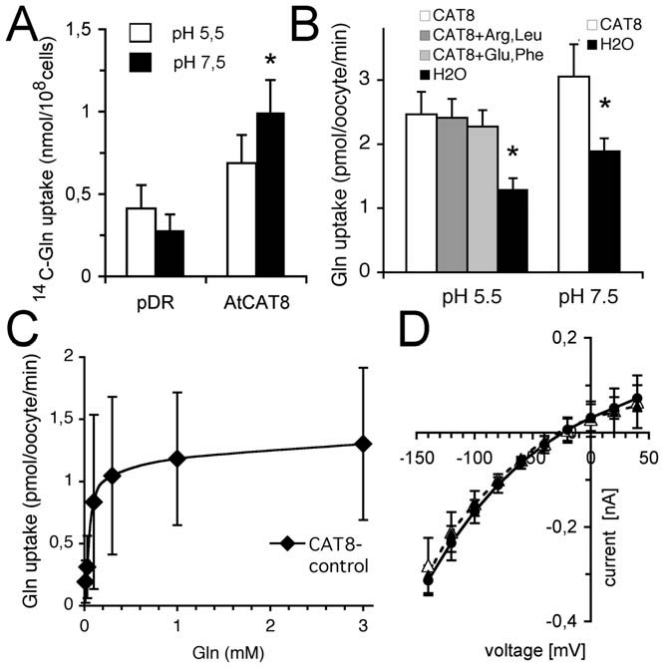
Functional characteristics of CAT8 in yeast and oocytes. (A) Uptake rates in 22Δ8AA yeast with 1 mM Gln. White bars: pH 5.5, black bars pH 7.5 (n = 4). The uptake by CAT8 was marginally significant at the p<0.05 level at pH 7.5 (*), using the Mann-Whitney test. (B) Uptake rates in oocytes at 1 mM Gln at pH 5.5 and pH 7.5. In the indicated experiments, Arg and Leu, or Glu and Phe, were added at 2 mM as competitors (n = 3).The uptake by CAT8 was significant at the p<0.05 level (Mann-Whitney test). (C) Concentration dependence of Gln uptake by CAT8. Uptake from water-injected oocytes was subtracted from CAT8-injected oocytes at pH 7.5. A linear background uptake was observed in both oocyte batches. (D) Current-voltage relations of CAT8-expressing oocytes in the absence (black circle) and presence (open triangle) of 1 mM Gln at pH 5.5 (n = 4, solid lines). The dashed line corresponds to the current-voltage relation of water-injected control oocytes from the same batch (closed triangles). Similar data were obtained at pH 7.5.

Using a promoter-GUS reporter construct, prominent CAT8 expression had previously been identified specifically in root tips and other rapidly dividing tissue [16]. Yeast expressing CAT8 were more sensitive to the structural Gln/Glu analog L-methionine sulfoximine (MSX), suggesting that CAT8 might transport Gln in yeast [16]. Thus, from the expression pattern and function, CAT8 was a candidate for part of the Gln fluxes detected above in root tips. Transport of Gln by CAT8 was then directly tested in the heterologous host yeast. The yeast strain 22Δ8AA, which lacked eight endogenous amino acid uptake systems [7], was used. An increased ^14^C-labeled Gln uptake by CAT8 was observed, but in contrast to other plant amino acid transporters, this uptake was rather weak and the uptake was more pronounced at pH 7.5 (Figure 4A).

The transport was then characterized in more detail in *Xenopus* oocytes. CAT8-expressing oocytes imported more ^14^C-Gln than water-injected controls (Figure 4B), which was similar at acidic and neutral pH. Furthermore, several other amino acids did apparently not compete for transport, which indicated that this transporter was rather specific for Gln (Figure 4B). A linear background uptake was observed in control oocytes, whereas the import by CAT8 saturated at very low concentrations. The Gln uptake by CAT8 was half maximal at ∼100 µM (Figure 4C). Interestingly, CAT8-expressing oocytes did not show Gln-dependent ionic currents, although they imported ^14^C-Gln (Figure 4B). Such currents were expected for a Gln/H^+^ co-transporter. The current-voltage relations of CAT8-expressing oocytes were similar to non-expressing controls (Figure 4D). There was no additional current by Gln on top of the inevitable background conductance, neither at pH 5.5 (Figure 4D), nor at pH 7.5. This strongly indicated that CAT8 was a H^+^-independent Gln facilitator.

### Sub-Cellular Localization of CAT8

**Figure 5:**
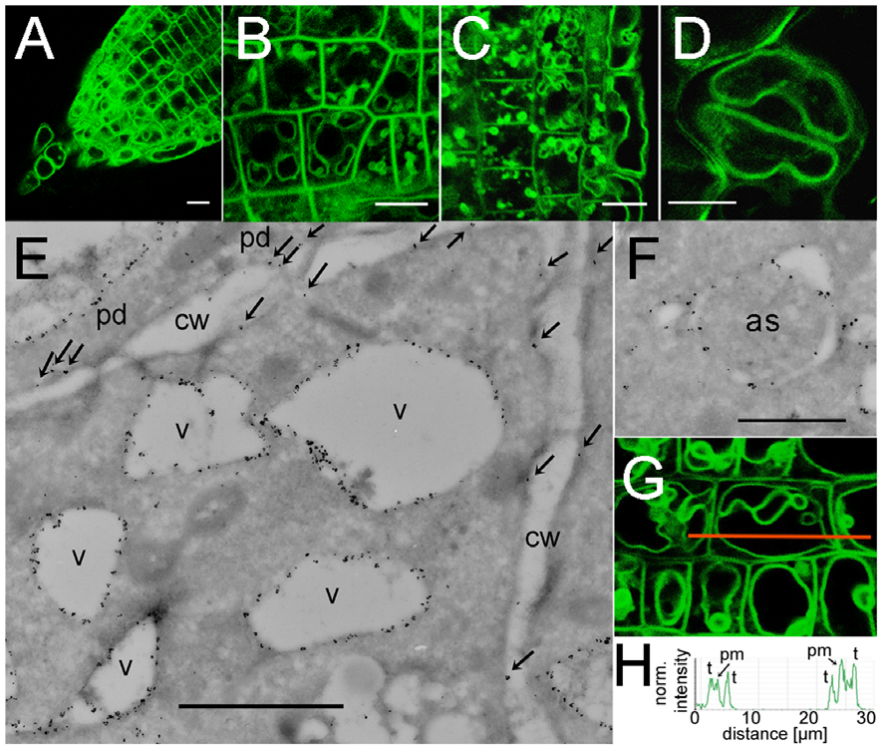
Sub-cellular localization of CAT8 expressed from the endogenous promoter in the plasma membrane and the tonoplast. (A–D) Subcellular localization of pCAT8::CAT8-GFP (A) in the root tip, (B) in the meristematic zone, (C) from the center to epidermal cells, (D) and guard cell. Scaling bars: 10 µm. (E) Ultrastructural analysis of pCAT8::CAT8-GFP plants with transmission electron microscopy and immunogold labeling using the GFP antibody. Tonoplast and plasma membrane localization of CAT8 is visible as black dots. The plasma membrane localization was highlighted by small arrows. Vacuole, v; plasmodesmata, pd; cell wall, cw. Scaling bar: 100 nm. (F) Immunogold labeling of CAT8-GFP in membranes of autophagocytotic structures (as). Scaling bar: 100 nm. (G, H) Quantitative analysis of the fluorescence intensity in plasma membranes and the tonoplast along the orange line. Fluorescence intensity histograms indicate similar fluorescence strength in the membranes.

GFP-labeled CAT8 was expressed from the endogenous promoter. CAT8 stained the plasma membrane, intracellular vesicles and the tonoplast (Figure 5A–D). Interestingly, the relative strength of the tonoplast to plasma membrane labeling somewhat differed with respect to the cell type. In the central cells close to the tip, which had only small vacuoles, the plasma membrane staining was most pronounced (Figure 5B). In contrast, in more basal epidermal root cells with large vacuoles, the tonoplast staining was more pronounced than that of the plasma membrane (Figure 5C). The fractions of CAT8-GFP at the tonoplast and the plasma membrane were estimated from fluorescence intensity histograms, along a line drawn through several cells (Figure 5G,H). This analysis showed that most central cells had an about equal intensity distribution in both membranes, but a higher tonoplast fraction was typically observed in epidermal cells (Figure 5C,D).

The dual labeling of the plasma membrane and the tonoplast was also supported by transmission electron micrographs from freeze-dried tissue (Figure 5E). In the cells shown, the immuno-gold-labeling of CAT8 was most strongly detected at the tonoplast, but also at the plasma membrane (Figure 5E, arrows). Furthermore, small vesicles that contained double membranes, reminiscent of autophagocytotic structures, were positive for CAT8 (Figure 5F).

**Figure 6:**
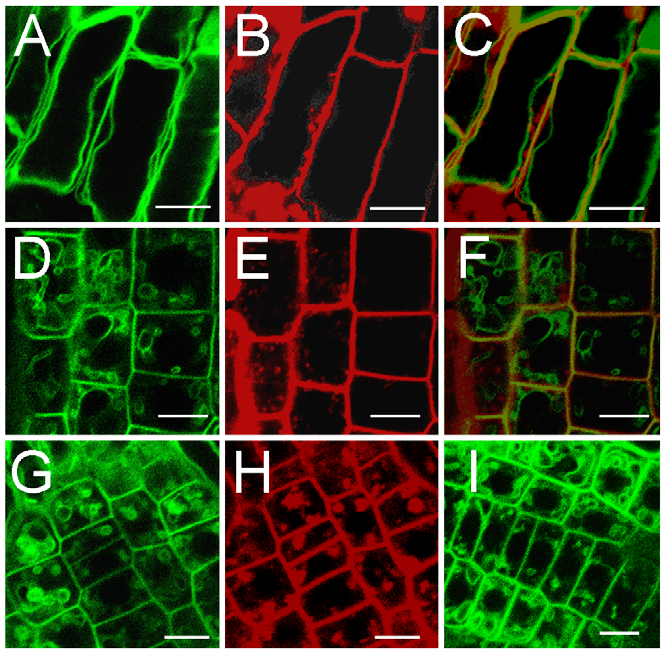
Localization of CAT8 to the plasma membrane and the tonoplast in the root tip. 35S::CAT8-GFP (A,D) and co-localization with FM4-64 (B,E, 15 min.) in the plasma membrane, but not in early endocytic vesicles, overlay (C,F). (G) Partial co-localization with FM4-64 (red, H) in 35S::CAT8-GFP plants after 2 h of BFA treatment. (I) Sub-cellular localization of 35S::GFP-CAT8 in the apical root zone. Scaling bars: 10 µm.

Since it remained possible that the subcellular distribution between tonoplast and plasma membrane depended on the protein expression level, CAT8-GFP was also expressed from the 35S promoter. 35S plants showed a highly similar cellular fluorescence pattern in the root tip as plants expressing the CAT8-GFP from the endogenous promoter (Fig. 6).

In co-labeling experiments with the membrane dye FM4-64, the plasma membrane was rapidly co-stained (Figure 6B,E). A few minutes after the addition of the lipophilic dye, early vesicular endocytotic vesicles were also labeled, in addition to the plasma membrane (Figure 6B,E). CAT8-GFP co-labeled the plasma membrane, but not the early endocytotic vesicles (Figure 6C,F). In the presence of the trafficking inhibitor Brefeldin A (BFA), the CAT8-labeled structures co-localized partially with FM4-64-labeled aggregates in the cell (Figure 6G,H). The fluorescent pattern was identical irrespective whether GFP was added at the C-terminus (Figure 6A,D,G) or at the N-terminus (Figure 6I).

### Deletion and Over-Expression of CAT8

**Figure 7:**
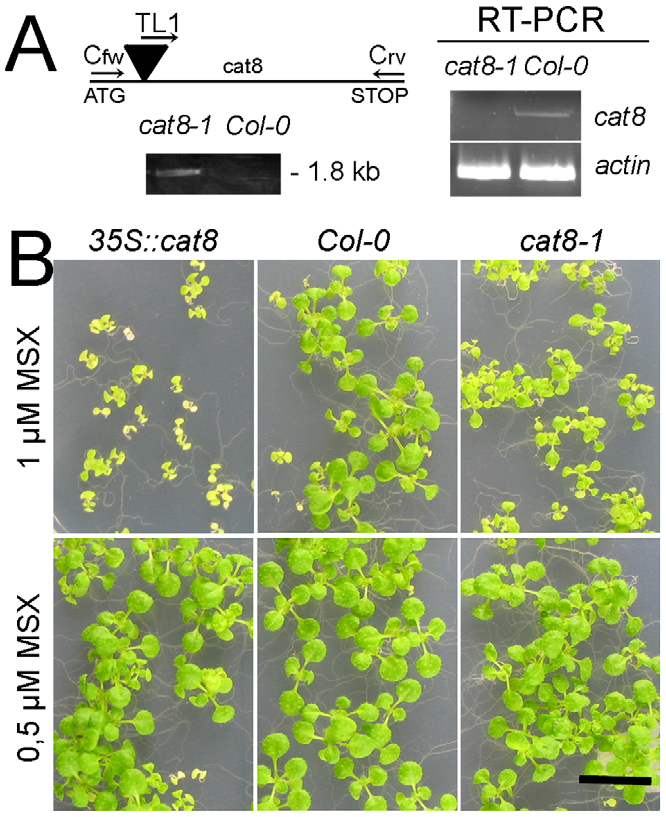
Isolation and characterization of *cat8-1* and 35S::CAT8 overexpressor lines. (A) Schematic drawing of the position of the T-DNA insertion (black triangle) and the used primers in the cat8 gene. Verification of the T-DNA insertion on genomic DNA (left panel) and identification of homozygous plants by RT-PCR confirmed by amplification of a 1739 bp fragment (right panel). Actin served as control. In all cases, 30 cycles were used. (B) Growth on 1 µM MSX (upper panel) and 0.5 µM MSX (lower panel). From left to right: *35S::CAT8*, wild type, *cat8-1*.

A T-DNA insertion line was obtained from the Salk collection and homozygous plants were isolated by genomic PCR and segregation analysis. The lack of the transcript was verified by RT-PCR (Figure 7A). Furthermore, homozygous *CAT8ox* lines expressing CAT8-GFP ectopically under the 35S promoter were isolated. These plants were germinated on agar medium that contained 50 µM or 1 mM Gln as nitrogen source, but no differences in growth were observed (*data not shown*). Similarly, no phenotypical differences compared to the wild type were observed with each proteinogenic amino acid at the same concentrations (*data not shown*). Plants were then grown on plates containing Murashige-Skoog medium supplemented with the toxic Gln/Glu analog N-methyl sulfoximine (MSX). MSX is a potent inhibitor of glutamine synthetase. The anionic form of MSX is phosphorylated at the sulfoximine nitrogen and then structurally resembles glutamate. This then blocks a transition state intermediate of the enzyme [28]. Lines over-expressing CAT8 (*CAT8ox*) were more sensitive to that drug (Figure 7B), while no or only minor differences were observed between knock-out and wild type plants (Figure 7B). This suggests that *CAT8ox* imported more MSX, which subsequently blocked glutamine synthetase.

## Discussion

### Fluorescent Reporters for Glutamine

Glutamine is an essential and important metabolite that efficiently promotes growth of *Arabidopsis* [3]. In addition to its nutritional role, Gln is supposed to have additional regulatory functions in several organisms [29]. In *Arabidopsis*, Gln is abundant in the root and xylem sap, as it is the primary product of ammonium assimilation [27]. The fluorescent reporter developed in this study was relatively specific for this important amide and may be a tool in the future to quantify this metabolite on the sub-cellular level. Furthermore, such sensors may be used to study local effects, such as those in the root tip as in this study. Local phenomena are often difficult to extract against a large background, and the total amino acid uptake in roots is known to be dominated by LHT1 [4], [5], although other transporters also contribute.

When reporter-expressing plants were superfused with Gln-containing solutions, very rapid changes in fluorescence were detected. After a stable fluorescence was obtained, the fluorescence decreased rapidly to initial levels after withdrawal of the substrate. The maximal fluorescence changes saturated, which either reflected the saturation of the uptake systems, or the intrinsic properties of the reporter. Because the affinities of typical plant amino acid transporters for Gln were in the sub-millimolar range (AAP1-AAP6: K_m_ = 0.06–1.2 mM) [4], [7], all these systems should operate at near maximal transport velocity at the Gln concentrations used. The saturation constants for the fluorescence may therefore reflect the saturation of the reporter, but further controls with other techniques appear necessary to fully resolve that issue.

When expressed in yeast, the uptake of amino acids by proton-coupled AAP or CAT amino acid transporters is completely inhibited by the protonophore CCCP (100 µM) [16], [30]. Surprisingly, the Gln-dependent FRET changes were not abolished with CCCP. At slightly basic pH, the fluorescence changes were also still large compared to the acidic pH, indicating that a different transport mechanism than in typical amino acid transporters was operating [16], [30].

It is possible that part of the large FRET changes at pH_ext_ 7.5 resulted from a higher sensitivity of the FRET reporter in these conditions, as the shift in pH_ext_ may have changed the pH_i_. In similar experiments, the cytosolic pH_i_ changed significantly during a pH_ext_ shift [22]. The FRET ratio of similar, CFP/YFP containing reporters however, was only slightly sensitive to the pH, while the substrate K_d_ was not [22]. Furthermore, the absolute ratio and the ratio change may differ at the different pH, mainly due to effects on the starting ratio. This may be explained by different relative sensitivities of the chromophores to pH (CFP was less affected than YFP) [22]. Furthermore, the FRET magnitude of an analogous reporter with identical chromophores was only weakly dependent on the pH [23]. Thus, although the amount of Gln transported was not linearly correlated with the maximal FRET responses at different external pH, the large FRET changes at pH 7.5 indicated significant transport, and were indicative of a proton-independent amide flux.

### A Proton-Independent Glutamine Facilitator in Plants

Correlative evidence suggested that part of the Gln transport was transported by CAT8, although the saturation properties of the FRET responses and CAT8 were clearly distinct. In previous yeast growth tests, CAT8 did not transport sufficient Lys, Cit, Pro, GABA, Asp and Glu to support growth on these substances [16]. However, yeast expressing CAT8 were more susceptible to the toxic Gln/Glu analog MSX [16], suggesting that CAT8 might transport glutamine. Indeed, yeast that expressed CAT8 had higher transport rates for ^14^C-glutamine than empty plasmid-transfected controls. The Gln uptake was, however, rather low and not pronounced at acidic pH. For a more detailed analysis, CAT8 was expressed in oocytes. Oocytes expressing CAT8 had increased ^14^C-glutamine uptake, which was similar at pH 5.5 and pH 7.5. Voltage-clamp experiments provided the proof for a H^+^-independent amide transport mechanism in CAT8 and explained its weak pH-dependence.

The closest homologs of CAT8 from plants are the cationic amino acid transporters CAT1 and CAT5, which preferentially transported Arg, Lys and His, but also some other amino acids, in a H^+^-coupled manner [16]. The more distant family member CAT6 showed H^+^-coupled amino acid transport, but had a completely different substrate spectrum [9]. CAT6 may be at least partially responsible for the H^+^-coupled Gln uptake observed in the FRET experiments, as it was also expressed in root tips [9]. Different biochemical properties, coupling mechanisms and substrate specificities have been reported in members from the same transporter super-families [31]. Since the Gln transport of CAT8 was not affected by a variety of other amino acids (Figure 4), this transporter may be relatively selective for Gln. Indeed, previous analysis using functional expression in yeast had already shown that other amino acids were not effectively transported by CAT8 [16]. However, it is still possible that further metabolites turn out as additional substrates of CAT8.

Plant lines stably expressing GFP-tagged CAT8 identified its dual targeting to vacuolar and plasma membranes, which is consistent with a recent tonoplast proteome analysis [18]. In protoplasts transiently expressing CAT8-GFP [16], the vast majority of cells was exclusively fluorescent at the plasma membrane, which was in contrast to the stably expressing lines. Furthermore, the localization in autophagosomes suggests that CAT8 may be involved in releasing Gln from these structures.

Plant growth assays using *cat8-1* and *CAT8ox* further supported a role of CAT8 in transporting Gln. These lines were not different on Gln, probably due to some redundancy in root amino acid transporters. However, *CAT8ox* plants were more sensitive to MSX, a toxic analog of Gln/Glu, which is likely explained by an increased uptake of MSX via CAT8 and subsequent impairment of glutamine synthetase. The combined data suggest that several, pH insensitive and pH sensitive systems are involved in transport of Gln across the plasma membrane in root tips.

The function of CAT8 in the tonoplast remains unclear, as an increased vacuolar import might be expected to increase the detoxification efficiency of roots, which was not observed. The very rapid efflux observed by the FRET reporters after removal of the substrate may argue against a large transport capacity across the vacuolar membrane, at least in the cells with the small vacuoles in the root tip. On the other hand, most amino acids have similar concentrations in the vacuolar lumen and in the cytoplasm [19], which is in accordance with a Gln facilitator function of CAT8 at the tonoplast.

The physiological role of CAT8 may thus be in equilibrating the tip tissue with Gln to supply all cells with assimilated nitrogen for growth. Alternatively, CAT8 may participate to exude Gln from the tip to attract beneficial micro-organisms, although the high affinity may argue against an efficient efflux capacity [32]. Preliminary data with labeled amino acids suggested that Gln efflux occurred in *Arabidopsis* [6]. Future work is required to identify whether CAT8 can account for the majority of the observed CCCP-independent FRET component in root tips, but this will require *CAT8* loss-of-function mutants in the *rdr6* background stably expressing the reporter. As the saturation properties of CAT8 differ from that of the Gln uptake detected by the FRET reporter, it is likely that other transporters also contribute.

## Materials and Methods

### Construction, Expression and Purification of Fluorescent Reporters

The constructs were entirely based on an initial construct containing a cassette of the QBP sequence, citrine, a mutant version of the enhanced yellow fluorescent protein (EYFP, Clontech) and the enhanced cyan fluorescent protein (ECFP) (Clontech). In this construct, the QBP sequence was split in two halves, and citrine was inserted between amino acid 98 and 99 of QBP [23]. The QBP-ECFP linker was shortened by the terminal 3 codons of QBP (amino acids TEP), and the neighboring glycine was exchanged to arginine, to reduce the flexibility of the linker. The ECFP sequence in the origignal construct was already shortened by four codons at the amino-terminus and citrine was shortened by six codons at the 5′ end and by ten codons at the 3′ end. The mutations threonine at position 70 replaced to alanine (T70A) and aspartate to asparagine (D157N) in QBP have been described [23]. All modifications were introduced by PCR and verified by sequencing.

The constructs were inserted into pET41 (Novagen) and the plant expression vector pPTkan. pET41 constructs were transferred into *E. coli* BL21(DE3)Gold (Stratagene) using heat shock. Cells were grown for 24h at 20°C to an OD of 0.6–0.8, induced with 0.2 mM IPTG and grown for 2 days at 20°C in the dark. Cells were harvested by centrifugation, resuspended in 20 mM Tris·Cl, pH 8.0, and disrupted by ultrasonication. Recombinant proteins were purified by His-Bind affinity chromatography resin (Novagen). Binding to the resin was performed at 4°C for 2 h and then washed with 20 mM Tris·HCl. The columns were incubated overnight on ice and subsequently washed with 20 mM Tris·HCl containing 20 mM imidazole at pH 8.0, and eluted with 200 mM imidazole in Tris·HCl, pH 8.0.

### In Vitro Characterization of the Reporters

Ligand binding curves were obtained from purified protein after overnight storage at 4°C. A monochromator microplate reader (Safire) was used to determine substrate binding affinities. Purified proteins were diluted in 20 mM Tris·HCl buffer (pH 8.0, final concentration of 11 mM imidazole). The excitation filter was 433/12 nm; emission filters for ECFP and Citrine were 475/12 and 528/12 nm, respectively. The apparent *K_d_* of each construct to different ligands was determined by fitting the data with a simple binding isotherm: I = (*r*−*r*_min_)/(*r*_max_−*r*_min_) = [L]/(*K_d_*+[L]), where [L] is the ligand concentration, *r* the ratio, *r*_min_ the minimum ratio in the absence of ligand and *r*_max_ the maximum ratio with saturating ligand. A Hill coefficient of n = 1 was determined in all measurements with the equation S = (*n*[L]*_n_*)/(*K*_d_+[L]*_n_*). Measurements were performed with at least three independent protein extracts. The fluorescence resonance energy transfer (FRET) ratio was determined as the fluorescence intensity at 528 nm divided by the intensity at 475 nm.

### Live Cell Imaging

Metabolite imaging was performed on a fluorescence microscope (DMIRB, Leica) with a cooled charge-coupled device camera (Sensys Photometrics, Tucson, AZ) and 40× objective. Dual emission intensity ratio was recorded by using METAFLUOR 4.5 software (Universal Imaging, Media, PA) with 436/20 excitation, two emission filters (480/40 for ECFP and 535/30 for EYFP) and a neutral density filter (1% or 5% transmission) on the excitation port. For the FRET ratio calculation, a focal spot with the highest fluorescence containing several cells was chosen. Plants were positioned in a small recording chamber that was initially built for electrophysiological oocyte recording and rapid solution change. The solution was supplied by gravity flow and was sucked from the chamber using a needle positioned on top of the solution level by a vacuum pump. Plants were fixed with medical adhesive (Hollister). The nutrient solution buffered to pH 5.5 or pH 7.5 with 5 mM MES (adjusted with TRIS) was used as recording medium. The complete exchange of the solution within the chamber was completed within a few seconds. Recordings were done on 6–10 day old seedlings. Even in the *rdr6* background, not all fluorescent plants responded to the superfusion with Gln (∼28%). The reason for that remains unclear. CAT8-GFP fusion constructs were imaged using a Leica confocal microscope.

### Ultrastructural Analysis

The transmission electron micrographs were obtained as in [33]. Briefly, immunogold labelling was performed on ultrathin (80–100 nm) thawed Tokuyasu cryosections of formaldehyde (8%, 2h) fixed and sucrose-infiltrated (2.1 M) root tips using rabbit anti-GFP serum (1∶250, 60 min; (Abcam)) and silver-enhanced (HQ Silver, 8 min; Nanoprobes) goat (Fab′) anti-rabbit IgG coupled to Nanogold (1∶50; No 2004, Nanoprobes).

*Plant selection, expression, transformation and growt*h The reporter sequences were shuttled into the plant expression vector *pPTbar*, a derivative of the *pPZP212* vector [34] containing the BASTA resistance, a *35S*-promoter and a *rbcs*-terminator. The *rdr6* mutant was obtained from the Arabidopsis stock center and plants were transformed using the GV3101 agrobacterium strain by flower spraying. Seeds were germinated in the dark for 4 days on agar plates (0.8%) containing minimal nutrient medium with 100 µM NH_4_NO_3_ as nitrogen source. Selection for fluorescent transgenic plants was done by the addition of 2 µM MSX to the plates and the most strongly expressing plants were selected under the fluorescent microscope. Plants from the T2 and T3 generation were used for Gln imaging.

GFP was cloned in frame to the complete CAT8 sequence (At1g17120) either at the N- or C-terminus, to generate translational fusions. In the C-terminal fusions, the STOP codon was removed by PCR. For both constructs, the 35S promoter from caulifower mosaic virus was used to drive expression. Another construct contained the endogenous genomic promoter region of 1832 bp in a 3.8 kb genomic fragment, that ranged by 758 bp into the neighboring gene (At1g17110). For all GFP constructs, pictures of homozygous T2 plants are shown that were grown on Murashige-Skoog (MS) medium (Duchefa). Mendelian segregation analysis indicated the presence of a single T-DNA insertion.

The mutant *cat8-1* was obtained from the Salk collection (SALK_023964). Segregation of homozygous plants carrying the T-DNA insertion was confirmed on genomic level by PCR, using the left border primer TL1 and the CAT8-specific reverse primer C_rv_: CCTATGATGTAGCTCATCAGCC. Lack of the transcript in *cat8-1* was verified by RT-PCR using C_rv_ and C_fw_: CTCggtaccATGATCCCTGCTTCAATGGAG primers.

### Yeast Transformation, Selection, and Transport Measurements

The yeast strain 22Δ8AA [7] was transformed with pDR vector containing the cDNA of CAT8 or empty vector pDR196 by heat shock. Transformants were selected on yeast nitrogen base (YNB) media without uracil, supplemented with 0.5 g/liter ammonium sulfate. Yeast cells were grown to logarithmic phase harvested at OD_600_ of 0.5, washed, and resuspended in ice cold buffer (50 mM KP_i_, pH 5.0, 0.6 M sorbitol) to a final OD of 5. One hundred microliters of cells were pre-incubated with 10 µl of 1 M glucose (30°C, 5 min). Uptake was initiated by the addition of 110 µl of radioactive substrate mixture, containing L-[^14^C]glutamine (11.2 GBq/mmol, Amersham Biosciences). Linear uptake rates were determined over 5 min. Samples were removed after 1,2,3 and 5 min, transferred to 4 ml of ice-cold buffer, filtered on glass fiber filters, and washed twice with 4 ml of buffer. Radioactivity was determined by liquid scintillation spectrometry. Endogenous uptake activity of yeast transformed with empty vector pDR196 was measured in parallel, but was not subtracted.

### Oocyte Expression, Uptakes and Electrophysiology

Oocyte handling, electrophysiology and uptakes were essentially done as described previously [7]. The recording and uptake solutions contained 100 mM NaCl, 2 mM MgCl_2_, 1.8 mM CaCl_2_, 5 mM MES adjusted to pH 5.5 or pH 7.5 with TRIS (tris(hydroxymethyl)aminomethane).

#### Abbreviations

The abbreviations used are: GUS, β–glucuronidase; GFP, green fluorescent protein; CAT, cationic amino acid transporter; QBP, glutamine binding protein; Gln, glutamine; Arg, Arginine; Lys, Lysine; His, Histidine; CCCP, carbonylcyanide 3–chlorophenylhydrazone; AAP, amino acid permease; FRET, Förster resonance energy transfer; MSX, N-Methyl sulfoximine; RT-PCR, reverse transcription-polymerase chain reaction, MES, 2-(N-morpholino)ethanesulfonic acid.
